# Dataset on the differential metabolite composition of ripe and green fruit coats of *Solanum mauritianum*

**DOI:** 10.1016/j.dib.2026.112829

**Published:** 2026-05-07

**Authors:** Abraham Goodness Ogofure, Ezekiel Green

**Affiliations:** a Department of Biotechnology and Food-Technology, Faculty of Science, University of Johannesburg, South Africa; b Molecular Pathogenic and Molecular Epidemiology Research Group (MPMERG), Department of Biotechnology and Food-Technology, Faculty of Science, University of Johannesburg, South Africa

**Keywords:** LC-QTOF-MS/MS, Secondary metabolites, Fruit maturation, *Solanum mauritianum*, Comparative metabolomics, Phytochemistry

## Abstract

This data article presents a comprehensive comparative metabolomic dataset characterizing the differential secondary metabolite profiles of ripe and green fruit coats of *Solanum mauritianum* Scop., an invasive species with significant ethnopharmacological relevance. The dataset complements the research article published by Ogofure et al [1] in Scientific Reports, which focused on ripe fruit components and identified promising antibacterial and anticancer properties. The current dataset addresses a critical research gap by providing untargeted LC-QTOF-MS/MS metabolomic profiling data comparing ripe and green fruit coats, revealing maturation-dependent biochemical transformations. A total of 35 secondary metabolites were putatively annotated (MSI Level 2) across both maturation stages, with detailed information on retention times, accurate mass measurements, and MS/MS fragmentation patterns. The ripe fruit coat exhibited 17 unique metabolites, while the green fruit coat contained 7 unique metabolites, with 11 metabolites shared between both stages. This dataset provides valuable insights into the ontogenic variation in phytochemical composition and offers a foundation for understanding the biosynthetic pathways active during fruit maturation.

Specifications Table

**Table:** 

Subject	Health Sciences, Medical Sciences & Pharmacology
Specific subject area	Plant metabolomics, Phytochemistry, Natural product chemistry
Type of data	Tables, Figures (Venn diagrams, Bar charts), LC-QTOF-MS/MS chromatograms
Data collection	The green fruit and ripe fruit coats from fresh *S. mauritianum* were collected, separated, surface-sterilized, dried and extracted using chloroform-methanol (1:1 v/v). Crude extracts were analyzed using untargeted metabolomic profiling. Peak detection, retention time alignment, and compound annotation were performed using Bruker Compass DataAnalysis software and multiple spectral databases (KEGG, PubChem, ChemSpider, MetFrag, ChEBI). Metabolites were annotated and evaluated in R-studio.
Data source location	University of Johannesburg, Doornfontein Campus, Johannesburg, South Africa
Data accessibility	Data are available in Figshare at https://figshare.com/s/44bddec519e5116967c1
Related research article	Ogofure AG, Sebola T, Green E. Antibacterial and anticancer properties of Solanum mauritianum fruit components analyzed using LC-QTOF-MS/MS. Scientific Reports. 2025;15:16,698. https://doi.org/10.1038/s41598–025–01348-w

## 1 Value of the Data

• This dataset provides the first comprehensive comparative metabolomic analysis of ripe versus green fruit coats of *S. mauritianum*, filling a critical knowledge gap identified in a previous publication [1]. Understanding the maturation-dependent metabolite changes is essential for optimizing harvest timing for bioactive compound extraction.
• Natural product chemists, pharmacologists, and drug discovery researchers can utilize this dataset to identify stage-specific bioactive compounds and prioritize metabolites for isolation, structural elucidation, and bioactivity screening. The differential metabolite profiles may guide targeted extraction strategies.
• The data revealed possible ontogenic variation in specialized metabolite production and contributes to the growing body of metabolomic data on invasive plant species with medicinal potential. It can be integrated with other omics datasets (transcriptomics, proteomics) for systems biology approaches to understanding plant chemical defense mechanisms and ethnopharmacological applications.
• The high-resolution MS/MS fragmentation patterns and retention time data provided can serve as reference standards for metabolite annotation in other *Solanum* species studies, facilitating comparative metabolomics across the Solanaceae family.

## 2 Background

*Solanum mauritianum* Scop. (also known as bugweed or woolly nightshade) is an invasive plant species with multiple documented ethnopharmacological uses across several continents [1,2]. Despite its widespread distribution, medicinal properties, and traditional applications, the systematic investigation of its phytochemical composition remains limited, particularly regarding the developmental variations in secondary metabolite production. In a recent publication, Ogofure et al [1] conducted comprehensive LC-QTOF-MS/MS metabolomic profiling and bioactive screening of the fruit components in *S. mauritianum.* The crude extract was reported to demonstrate significant antibacterial activity against pathogens of public health importance and selective anticancer activity against U-87 MG glioblastoma cells. The ripe fruit coat emerged as the part with most bioactive components, exhibiting the richest phytochemical diversity with 15 unique metabolites and demonstrating potent cytotoxic effects.

However, a critical limitation of that study was the absence of comparative metabolomic data from green (unripe) fruit coats. This gap prevented a comprehensive understanding of maturation-dependent biochemical transformations and optimal harvest timing for bioactive compound extraction. Plant secondary metabolism undergoes dynamic shifts throughout fruit development, marked by substantial quantitative and qualitative changes in metabolite profiles as fruits progress from immature to fully ripe stages [[3], [4], [5]]. These ontogenic variations reflect substrate availability, complex biosynthetic pathway regulation, and various physiological roles of secondary metabolites in fruit defense, ripening, and seed dispersal [[6], [7], [8]].

It is important to note that the ripe fruit coat (RFC) LC-QTOF-MS/MS data presented in this dataset article were generated from the same bulk plant material and analytical run as reported in the related research article [1]. The present dataset article serves as the primary data companion to that publication, extending the comparative analysis to include green fruit coat (GFC) data alongside RFC data, and providing a comprehensive RFC-versus-GFC metabolomic characterization beyond the scope of the original research article. The raw data files encompassing both maturation stages are deposited in Figshare and are referenced in this publication.

## 3 Data Description

**Fig. 1:**
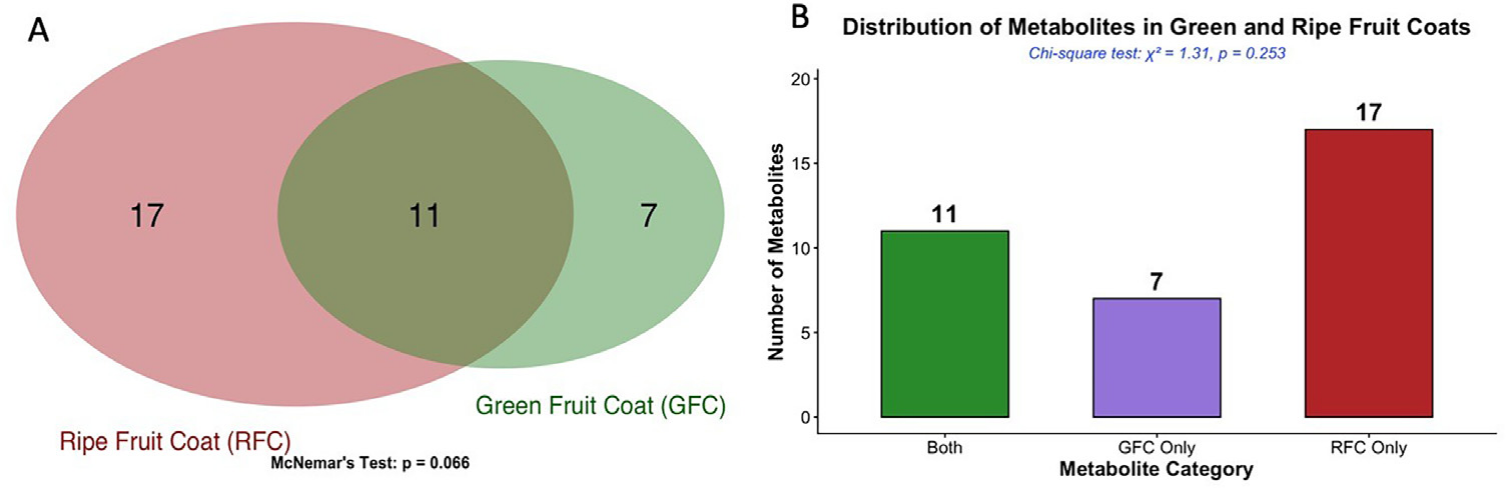
Distribution and overlap of metabolites in green and ripe fruit coats of *S. mauritianum.* (A) Scaled Venn diagram illustrating the qualitative set relationships: the number of metabolites detected exclusively in the GFC, exclusively in the RFC, and those shared between both developmental stages. The diagram visually conveys the relative set sizes and degree of metabolite overlap. (B) Bar chart providing a quantitative summary of stage-specific and shared metabolite counts, which forms the basis for McNemar’s test (paired asymmetry assessment) and chi-square analysis (independence of distribution category and fruit coat stage). Together, both panels describe the metabolite distribution pattern at complementary levels of resolution*.*

The dataset comprises comprehensive LC-QTOF-MS/MS metabolomic profiles of ripe and green fruit coats of *Solanum mauritianum*, revealing the maturation-dependent variation in secondary metabolite composition between the ripe fruit coat (RFC) and the green fruit coat (GFC). A total of 35 distinct secondary metabolites were annotated across both developmental stages, providing insight into the biochemical complexity of the fruit coat tissues of this invasive species. The present study reports a comprehensive comparative metabolomic dataset that includes: (1) visualization of differential metabolite distribution using Venn diagrams and comparative charts; (2) annotated secondary metabolite profiles for both ripe and green fruit coats with detailed spectroscopic parameters; (3) classification of metabolites by chemical class; (4) high-resolution MS/MS spectra and chromatographic data; and (5) statistical comparison of metabolite abundance between maturation stages. This dataset complements the published research article by Ogofure et al [1] and provides a valuable resource for the scientific community interested in plant metabolomics, natural product chemistry, drug discovery, and developmental biochemistry of Solanaceae species. Several of the annotated secondary metabolites, particularly steroidal alkaloids and flavonoid derivatives, have been previously associated with antimicrobial [1,9,10], cytotoxic [11], antioxidant, and plant defense functions in *Solanum* and related taxa [8,12,13], underscoring the biological relevance of the detected metabolite classes. The overlap and distribution of secondary metabolites detected in the green and ripe fruit coats of *S. mauritianum* are shown in Fig. 1. Eleven metabolites were common to both developmental stages, while 7 metabolites were detected exclusively in the green fruit coat and 17 were detected exclusively in the ripe fruit coat. The Venn diagram (Fig. 1A) illustrates the shared and stage specific metabolite composition, while the bar chart (Fig. 1B) provides a quantitative summary of these categories. McNemar’s test did not reveal strong asymmetry under the applied exploratory test in paired metabolite presence between the two fruit coat stages (*p* = 0.066). Similarly, Chi-square analysis showed no significant association between fruit coat stage and metabolite category (*χ² = 1.31, p =* *0.253*), indicating that the observed differences in shared and unique metabolites were not statistically significant. Nevertheless, the numerical enrichment of ripe-specific metabolites suggests progressive metabolic diversification during fruit maturation, a pattern consistent with reported ontogenic shifts in secondary metabolism in several Solanaceae plants [5,8,[14], [15], [16]]. Such maturation-linked metabolic remodelling may reflect increased investment in defensive or signalling compounds during the later stages of fruit development [8]. These findings have significant implications for optimizing harvest timing when targeting specific bioactive compounds.

This differential distribution pattern indicates dynamic metabolic reprogramming during fruit maturation. The higher number of ripening-specific metabolites (17) compared to green-specific metabolites (7) suggests that ripening is associated with the activation of additional biosynthetic pathways or the accumulation of mature-stage defensive compounds. These findings have significant implications for optimizing harvest timing when targeting specific bioactive compounds. It should be noted that the earlier Scientific Reports article by Ogofure et al [1] reported 15 RFC-specific metabolites, whereas the present dataset reports 17. This difference reflects updated curation during dataset consolidation: two metabolites that were detectable in the raw LC-QTOF-MS/MS data and met MSI Level 2 annotation criteria that were inadvertently omitted from the earlier summary of RFC-specific features during reporting. All metabolite annotations were re-checked and harmonized across both maturation stages in this present study using consistent criteria and database cross-referencing, and the counts reported here (RFC-only = 17; GFC-only = 7; shared = 11) represent the final curated dataset.

**Fig. 2:**
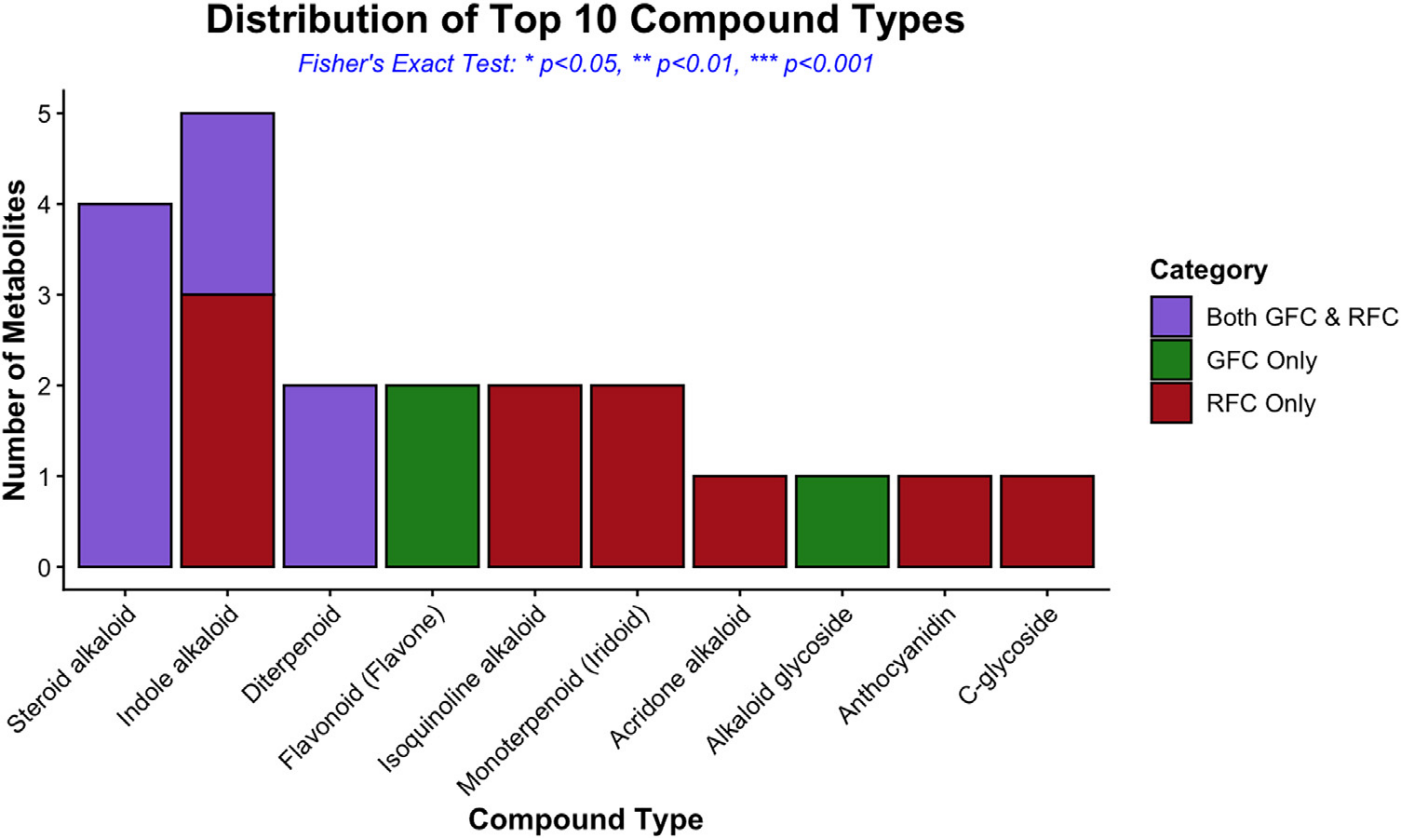
Distribution of the top 10 compound types detected in ripe fruit coat (RFC) and green fruit coat (GFC) samples.

The classification of all annotated metabolites into 10 major chemical classes is shown in Fig. 2. Alkaloids represent the predominant class, accounting for 57.14% of total metabolites (12 compounds), followed by terpenoids at 19.04% (4 compounds). This alkaloid dominance is characteristic of the Solanaceae family and consistent with the known biosynthetic capabilities of *Solanum* species. Glycosides and other classes of compounds were also present, albeit in smaller proportions. Specifically, the 12 alkaloids (distinct from the four steroidal alkaloids classified separately under the “Steroid alkaloid” class in Fig. 2) comprised five indole alkaloids (ibogamine, α‑ergocryptine, 10‑deoxysarpagine, ibogaine, and lysergic acid), two isoquinoline alkaloids (berberastine and thalicarpine), one pyridine alkaloid (anatabine), one quinazoline alkaloid (glycophymoline), one quinoline alkaloid (cusparine), one quinolizidine alkaloid (lycocernuine), and one acridone alkaloid (furofoline I) [10,13]. The four steroidal alkaloids were imperialine, solasodine, solasonine, and α‑solanine, compounds well known in Solanum species for their steroidal saponin‑type biological activities. The terpenoids in Fig. 2 included the diterpenoids callicarpone and montanol; the monoterpenoid iridoids genipin and loganin; the sesquiterpenoid guaianolide absinthin; and the triterpenoid tingenone, each assigned to its respective subclass category. Steroidal and indole alkaloids detected in this study (such as the solasodine-related compounds) are widely reported to contribute to plant chemical defense against herbivory and microbial attack, and several have documented pharmacological activities including anticancer, antimicrobial, and anti-inflammatory effects [8,10,13,17]. Comparative analysis of the top ten compound classes between green and ripe fruit coats showed no statistically significant difference in class level distribution, as indicated by a Wilcoxon signed rank test (*p* = 0.668). In addition, Fisher’s exact test revealed no significant association between compound class and fruit coat developmental stage, indicating that the overall chemical class composition remained comparable between green and ripe fruit coats despite differences in individual metabolites. This suggests that fruit maturation in *S. mauritianum* may involve qualitative reshuffling within conserved biosynthetic classes rather than wholesale class-level shifts, an observation that may inform future targeted metabolite discovery and stage-specific harvesting strategies. It should be noted that the present untargeted workflow was optimized for the detection of mid-polar to non-polar secondary metabolites. Thus, highly polar primary metabolites, such as simple sugars and organic acids, are likely underrepresented due to the chloroform:methanol extraction system and the reversed-phase LC-ESI-QTOF analytical configuration. Consequently, the current dataset is intended to describe maturation-dependent variation in specialized (secondary) metabolism rather than primary carbohydrate metabolism.

The LC–MS workflow was designed for qualitative profiling rather than absolute or relative quantification, Table 1 focuses on compound identification (class, formula, diagnostic fragments and presence/absence by tissue and maturation stage) and does not include peak intensity values. The metabolite class distribution between ripe and green fruit coats of S. mauritianum (Table 1) revealed interesting patterns of class-specific accumulation across maturation stages, indicating significant metabolic reprogramming during fruit ripening. The ripe fruit coat exhibits substantially higher diversity in alkaloid content compared to the green fruit coat. Several steroidal alkaloids, including solasodine, solasonine, and α-solanine, were prominently detected in both developmental stages, underscoring their constitutive expression throughout fruit ontogeny. Conversely, the green fruit coat exhibits different alkaloid profiles, particularly enriched in quinolizidine and pyridine alkaloids, suggesting maturation-dependent transformation or de novo biosynthesis of specific alkaloid subclasses.

**Table 1:** Comparative metabolite profiling of ripe and green fruit coats of S. mauritianum showing class distribution, retention times, and mass spectrometric data.

S/N	Metabolite Name	Molecular Formula	Metabolite Class	Rt (min)	Err (ppm)	[M + H]+ (*m/z*)	Key MS/MS Fragment Ions (*m/z*)	Category
1	Furofoline I	C₁₆H₁₁NO₃	Acridone alkaloid	1.36	−10.1	266.0812	98.0381, 116.0467, 230.0637, 248.0703	RFC Only
2	Vicine	C₁₀H₁₆N₄O₇	Alkaloid glycoside	5.72	−8.8	305.1090	70.0467, 112.0615, 305.1069	GFC Only
3	Riccionidin A	C₁₅H₉O₆	Anthocyanidin	1.55	−9.4	286.0472	80.0308, 124.0138, 286.0453	RFC Only
4	Bergenin	C₁₄H₁₆O₉	C-glycoside	2.13	2.7	329.0867	120.0553, 166.0556, 264.0791, 310.0792	RFC Only
5	Scopoletin	C₁₀H₈O₄	Coumarin	6.61	−4.7	193.0495	77.0207, 90.0254, 103.0313, 118.0170, 133.0378, 161.0290, 193.0508	Both
6	Cardiospermin	C₁₅H₁₇NO₄	Cyanogenic glycoside	1.27	−12.6	276.1230	86.0678, 161.0375, 230.0987, 258.0900	Both
7	Callicarpone	C₂₀H₂₈O₄	Diterpenoid	12.57	5.1	333.2060	95.0633, 279.1858	Both
8	Montanol	C₂₀H₃₂O₅	Diterpenoid	16.64	−7.6	353.2323	81.0504, 261.1768	Both
9	Homoeriodictyol chalcone	C₁₆H₁₄O₆	Flavonoid (Chalcone)	5.14	−8.9	303.0863	105.0114, 195.0573, 285.0783,	RFC Only
10	Diosmin	C₂₈H₃₂O₁₅	Flavonoid (Flavone)	25.77	−4.4	609.1814	280.2147, 476.1454, 609.1812	GFC Only
11	Isoswertisin 2′'-rhamnoside	C₂₈H₃₂O₁₄	Flavonoid (Flavone)	27.34	−5.7	593.1865	461.1583, 533.1717, 593.1855	GFC Only
12	Theogallin	C₁₄H₁₆O₁₀	Glycoside	5.14	−7.8	345.0816	137.0334	RFC Only
13	Ibogamine	C₁₉H₂₄N₂	Indole alkaloid	26.28	−14.6	281.2012	83.0659, 133.0742, 221.1875, 245.1844, 263.1927	Both
14	α-Ergocryptine	C₃₂H₄₁N₅O₅	Indole alkaloid	10.11	−4.7	576.3180	85.0088, 157.0711, 253.1526	Both

15	10-Deoxysarpagine	C₁₉H₂₂N₂O	Indole alkaloid	12.60	−9.1	295.1805	93.0477, 151.0835, 277.1753	RFC Only
16	Ibogaine	C₂₀H₂₆N₂O	Indole alkaloid	26.38	−8.7	311.2118	81.0507, 99.0577, 153.0969, 279.1855	RFC Only
17	Lysergic acid	C₁₆H₁₆N₂O₂	Indole alkaloid	7.09	−3.3	269.1285	81.0515	RFC Only
18	Berberastine	C₂₀H₂₀N₂O₄	Isoquinoline alkaloid	6.21	−7.6	353.1501	110.0376, 136.0473, 182.0486	RFC Only
19	Thalicarpine	C₄₁H₄₈N₂O₈	Isoquinoline alkaloid	19.47	−3.9	697.3483	105.0471, 697.3305	RFC Only
20	Coniferyl alcohol	C₁₀H₁₂O₃	Monolignol	18.37	−4.4	181.0859	77.0192, 107.0612, 121.0745, 135.0880, 163.0787, 181.0869	GFC Only
21	Genipin	C₁₁H₁₄O₅	Monoterpenoid (Iridoid)	9.06	8.8	227.0914	68.0323, 86.0771, 114.0326, 159.0342, 227.1021	RFC Only
22	Loganin	C₁₇H₂₆O₁₀	Monoterpenoid (Iridoid)	3.17	−6.9	391.1599	128.0810, 193.0508, 230.1019, 307.1160, 355.1306, 391.1497	RFC Only
23	Hypercalin B	C₃₁H₃₄O₇ §	Phloroglucinol	18.88	−6.2	519.2377§	104.0832, 184.0388, 303.6993, 518.2485	RFC Only
24	Cannabielsoin	C₂₁H₃₀O₃	Polyketide (Cannabinoid)	26.00	1.5	331.2268	95.0627, 239.1933, 313.2189, 331.2300	GFC Only
25	Anatabine	C₁₀H₁₂N₂	Pyridine alkaloid	20.92	−16.8	161.1073	116.0392, 161.0641	Both
26	Eugenin	C₁₁H₁₀O₄	Pyrone – Chromone	19.46	−13.0	207.0652	105.0478, 161.0270, 176.0502	RFC Only
27	Glycophymoline	**C₁₂H₁₄N₂O₄**	Quinazoline alkaloid	7.28	6.8	251.1030	79.0361, 119.0268, 147.0150,	RFC Only
28	Cusparine	**C₁₉H₁₇NO₃**	Quinoline alkaloid	3.21	−8.8	308.1281	86.0768, 146.0893, 230.0990, 290.1121	RFC Only
29	Lycocernuine	**C₁₆H₂₆N₂O₂**	Quinolizidine alkaloid	24.42	−9.7	279.2073	81.0479, 173.0971, 209.1133, 261.1739, 279.1819	GFC Only
30	Absinthin	**C₃₀H₄₀O₆**	Sesquiterpenoid (Guaianolide)	27.97	−11.7	497.2901	89.0377, 133.0576, 184.0352	GFC Only
31	Imperialine	**C₂₇H₄₃NO₃**	Steroid alkaloid	8.69	−6.3	430.3316	70.0481, 157.0706, 253.1532, 430.2686	Both
32	Solasodine	**C₂₇H₄₃NO₂**	Steroid alkaloid	9.83	−6.5	414.3367	70.0479, 157.0712, 253.1525, 4142,751	Both
33	Solasonine	**C₄₅H₇₃NO₁₆**	Steroid alkaloid	8.39	−3.1	884.5002	70.0481, 884.3903	Both
34	α-Solanine	**C₄₅H₇₃NO₁₅**	Steroid alkaloid	8.64	−3.1	868.5053	129.0284, 253.1529, 868.3953	Both
35	Tingenone	**C₂₈H₃₆O₃**	Triterpenoids	31.85	−6.4	421.2737	421.2636	RFC Only

Note: Table 1 is intended as a structural/annotation summary. It lists the detected metabolites together with their classes, molecular formulas, key MS/MS fragment ions (*m/z*), and occurrence in each tissue/maturation stage. Signal intensities were used only to support peak detection and identification during data processing and were not retained or reported as quantitative abundance measures.

**Fig. 3:**
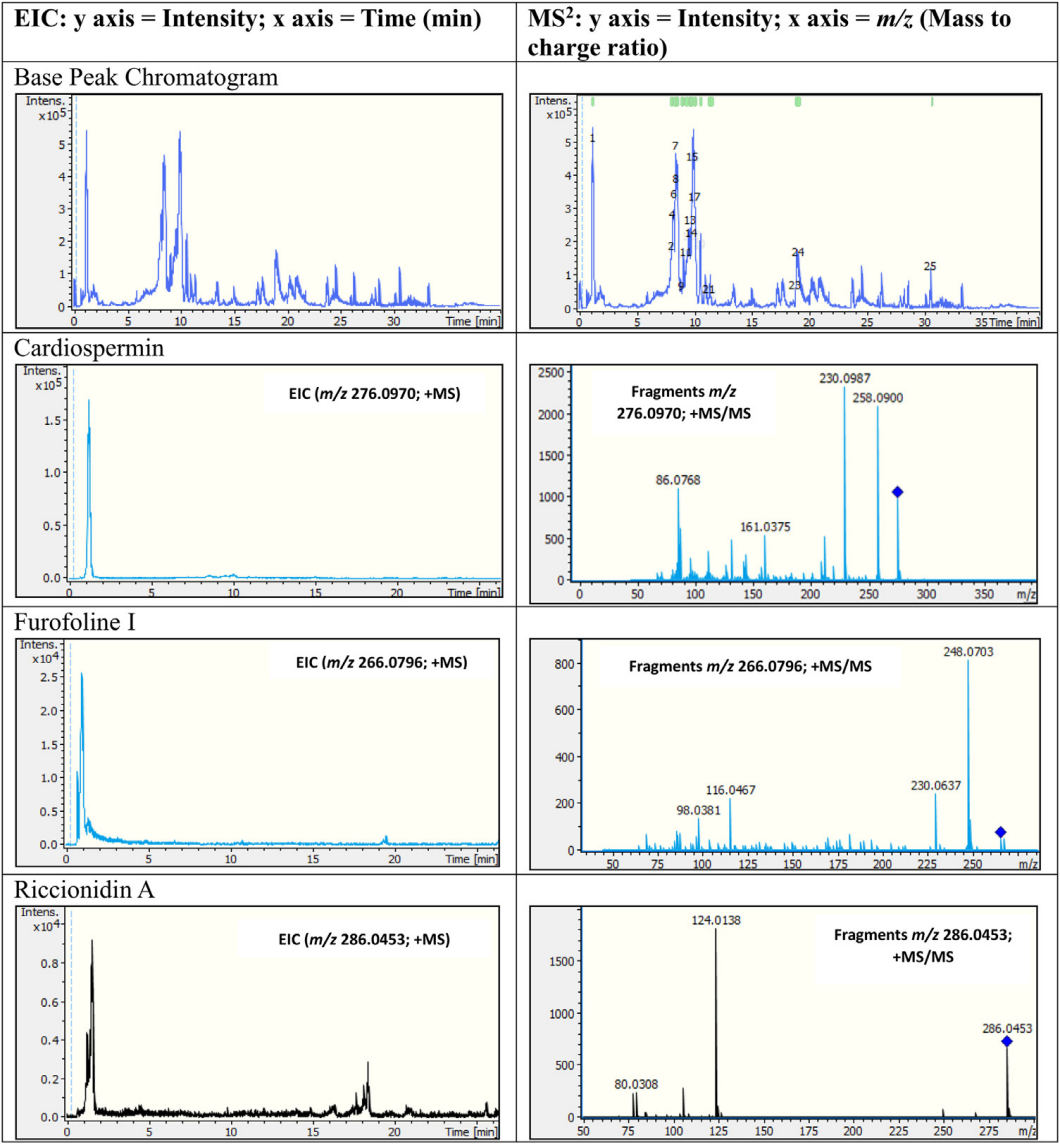
Representative base peak chromatograms (BPC) for ripe and green fruit coat of extracts of *S. mauritianum.* Please note that the BPC for the other annotated compounds is shown in supplementary Figures 1 and 2.

The representative base peak chromatograms (BPC) for ripe and green fruit coat extracts, illustrating the overall metabolite complexity and differences in chromatographic profiles between maturation stages is shown in Fig. 3. The chromatograms reveal distinct differences in peak patterns, intensities, and retention time distributions, providing visual evidence of differential metabolite composition.

## 4 Experimental Design, Materials and Methods

### 4.1 Sample collection and preparation

*S. mauritianum* Scop. fruits were collected from the University of Johannesburg's Doornfontein campus (S26 11 32.6 E28 03 28.9; approximately 1.753 m above sea level), South Africa, during the fruiting season (October 2019, corresponding to mid-to-late spring season). The species occurs abundantly at the collection site as an invasive weed, and the plant material was formally identified by a botanist, Prof. Annah Moteetee (Dean, Faculty of Science). A voucher specimen number (BTNPSP02) was assigned and deposited in the publicly accessible herbarium of the University of Johannesburg. Bulk fruit harvesting was performed from >20 individual plants to capture representative population-level metabolite diversity. Fruits were collected separately according to maturation stage into sterile 10-L containers in the field. Individual fruits were not counted because sampling was designed as bulk population harvesting, consistent with metabolomic survey studies of invasive species. The fresh fruits were categorized using predefined and standardized visual maturity criteria, with unripe fruits exhibiting uniformly green colouration, firm texture and without any pigmentation, whereas ripe fruits displayed uniform yellow colouration across the fruit surface with softened pericarp. The tissue designated ‘fruit coat’ in this study corresponds to the pericarp of *S. mauritianum* fruits, comprising the exocarp (epicarp) and mesocarp layers, which is distinct from the seed tissue. This outer fleshy layer was the sole tissue subjected to metabolomic profiling in both maturation stages. To ensure consistency, only the fruits which meet the full visual criteria were included in each maturity class, while the ambiguously intermediate fruits were excluded from the sampling strategy. Representative images of both maturation stages are provided in Supplementary Figure S1. For metabolomic analysis, three independent biological replicates were prepared for each maturation stage (*n* = 3) by randomly subsampling fruit from the bulk harvest, pooling the subsamples, and processing each replicate separately. Each replicate, therefore, represents a composite sample derived from multiple plants, providing a robust representation of the field population. For each maturation stage, fruit coats were manually separated from seeds using sterile scalpels. The separated fruit coats were thoroughly washed with sterile distilled water (at least 3 washes) to remove epiphytes and surface contaminants. Complete surface sterilization was achieved by brief immersion in absolute ethanol followed by 5 sterile distilled water rinses. We considered that several targeted secondary metabolites (such as phenolics and alkaloids) can exhibit partial solubility in polar solvents; thus, during washing, samples were visually inspected, and no visible pigment leaching was observed. While this suggests minimal compound loss, minor depletion of highly polar metabolites cannot be entirely excluded and is acknowledged as a methodological limitation. The sterilized samples were then dried at room temperature in a laminar flow hood, wrapped in aluminium foil, and stored in a desiccator until extraction.

### 4.2 Extraction procedure

The extraction methodology was adapted from previously published protocol of Ogofure et al [1] with specific optimization for comparative metabolomic analysis. Dried fruit coat samples were pulverized using a commercial blender to achieve uniform particle size distribution, enhancing extraction efficiency. For each maturation stage, 200 g of the powdered material was subjected to extraction. The extraction solvent consisted of a 1:1 (v/v) mixture of chloroform and methanol (HPLC grade), chosen for its broad polarity range capable of extracting both lipophilic and moderately polar secondary metabolites.

The extraction was performed using a 3000 mL capacity Schott bottle containing 2000 mL of the solvent mixture. Samples were extracted on an IKA® Rocker 3D basic (Inqaba Biotec, SA) for 48 h at room temperature to ensure complete metabolite extraction while avoiding thermal degradation of heat-sensitive compounds. Following extraction, the suspension was filtered through Whatman No 1 filter paper. The filtrate was concentrated using a VP30 LabTech rotary evaporator (Hopkinton, Massachusetts) at 45 °C under reduced pressure. To maximize extraction yield, the extraction process was repeated twice with fresh solvent on the same plant material. The combined crude extracts were pooled, concentrated to dryness, transferred to amber bottles, and stored in a desiccator under moisture-free conditions until LC-QTOF-MS/MS analysis.

### 4.3 LC-QTOF-MS/MS metabolomic profiling

#### 4.3.1 Sample preparation for LC-QTOF-MS/MS

The crude extracts were dissolved in HPLC-grade methanol (Merck, South Africa) at a concentration of 1 mg/mL. Complete dissolution was achieved through ultrasonication for 8–10 min using a bath sonicator. Following dissolution, samples were filtered through 0.22 μm PVDF (polyvinylidene fluoride) syringe filters directly into 1 mL capacity LC-MS autosampler vials. This filtration step removed particulate matter that could interfere with chromatographic separation or contaminate the mass spectrometer. HPLC-grade methanol without any analyte served as blank samples for system calibration and background correction. These blanks were analyzed under identical conditions as sample extracts to account for potential contributions from filter material or solvent impurities.

#### 4.3.2 Chromatographic conditions

Comprehensive analysis of secondary metabolites was conducted using an ultra-high-performance liquid chromatography system coupled with quadrupole time-of-flight mass spectrometry. The LC system comprised a Dionex UltiMate 3000 UHPLC (Thermo Scientific, Darmstadt, Germany) interfaced with a Bruker Compact™ QTOF mass spectrometer (Bruker Daltonics, Bremen, Germany). Chromatographic separation was achieved using reverse-phase UHPLC on a Raptor ARC-18 column (Restek, USA) with dimensions of 100 mm length × 2.1 mm internal diameter, packed with 2.7 μm particles and featuring 90 Å pore size. The mobile phase consisted of solvent A (0.1% formic acid in water, v/v) and solvent B (0.1% formic acid in acetonitrile, v/v). A gradient elution program was employed over 40 min: initial conditions of 5% B for 2 min, followed by a linear gradient to 95% B over 28 min, maintenance at 95% B for 5 min (isocratic hold), and rapid return to 5% B in 1 min for column re-equilibration. The flow rate was maintained at 0.3 mL/min throughout the analysis, and the injection volume was 5 μL. The autosampler was maintained at 10 °C, and the column oven temperature was set at 35 °C to ensure reproducible retention times and peak shapes.

#### 4.3.3 Mass spectrometry parameters

Mass spectrometric detection was performed using a Bruker Compact™ QTOF mass spectrometer equipped with an electrospray ionization (ESI) source operating in positive ion mode. The mass analyzer scanned from *m/z* 50 to 1600, providing comprehensive coverage of secondary metabolite molecular weight range. Key ESI source parameters were optimized as follows: capillary voltage of 4500 V, nebulizer pressure of 1.8 bar, dry gas flow rate of 8 L/min, and dry gas temperature of 220 °C. These parameters ensured efficient ionization and desolvation while minimizing in-source fragmentation. For tandem mass spectrometry (MS/MS), the instrument was configured with ionization energy of 4.0 eV and collision energy of 7.0 eV. Data acquisition was performed in centroid mode with a cycle time of 0.5 s. To ensure high mass accuracy throughout the analytical run, internal lock mass calibration was employed, minimizing mass drift and maintaining mass accuracy within ±5 ppm. It should be noted that no exogenous spike-in internal standard was added to the sample extracts at any stage of the sample preparation workflow. The lock mass calibration described above refers exclusively to instrument-level mass axis correction using a sodium formate calibrant solution infused via the dedicated lock-spray inlet of the Bruker Compact QTOF, which ensures mass measurement accuracy throughout the analytical run but does not serve as a quantitative internal standard for concentration normalization. The HyStar software version 2.10 (Bruker, Germany) controlled the entire LC-QTOF-MS/MS system, managed data acquisition, and facilitated real-time monitoring of analytical performance.

#### 4.3.4 Data processing and metabolite annotation

Raw LC-QTOF-MS/MS data files were processed using Bruker Compass Data Analysis software (version 4.3). Feature detection and mass peak extraction were conducted with stringent quality criteria: a signal-to-noise ratio threshold of 3:1, a minimum peak width of 5 scans, and a mass accuracy tolerance of ±5 ppm for both precursor and fragment ions. Peak deconvolution was performed using the recursive feature extraction module with Savitzky-Golay smoothing (polynomial order 2, window size of 7 points).

Baseline correction employed the rolling ball algorithm with a 10% noise band to enhance peak purity and minimize false positives from background noise or overlapping signals. Retention time alignment across all samples was conducted using a tolerance window of ± 0.2 min, ensuring reliable comparison of metabolite elution profiles between ripe and green fruit coat samples. This alignment step was critical for accurate comparative metabolomics, allowing identification of stage-specific metabolites versus those present in both maturation stages.

Metabolite annotation was performed through systematic cross-referencing of spectral data with multiple authoritative databases: Kyoto Encyclopedia of Genes and Genomes (KEGG), PubChem, ChemSpider, MetFrag, Chemical Entities of Biological Interest (ChEBI), and the National Institute of Standards and Technology (NIST) 2005 library. Putative identification was based on three complementary criteria viz; (1) accurate mass measurements of precursor ions; (2) MS/MS fragmentation patterns and diagnostic fragment ions; and (3) chromatographic retention time comparison with literature values. Metabolites were classified according to Level 2 confidence based on Metabolomics Standards Initiative (MSI) guidelines [18], reflecting putative annotation through spectral library matching without analytical standard confirmation. Compounds were retained for annotation only if they met all of the following criteria: mass accuracy within ± 5 ppm, MS/MS spectral similarity with cosine score ≥0.7 when compared to reference spectra, and retention time within ± 0.2 min of database or literature-reported values.

#### 4.3.5 Comparative metabolomics, experimental design and data visualization

Comparative analysis of metabolite profiles between ripe and green fruit coats was performed using R Studio (version 4.5.1) with specialized packages for metabolomics data analysis and visualization. The study followed a comparative metabolomic design in which fruit maturation stage (green fruit coat versus ripe fruit coat) was treated as the primary experimental factor. Three independent biological replicates were analyzed for each maturation stage (*n =* *3* per group), with each replicate representing an independently processed composite subsample derived from the bulk field collection. Metabolite presence and distribution between green fruit coat (GFC) and ripe fruit coat (RFC) of *S. mauritianum* were explored using a combination of descriptive visualization and inferential statistics. All statistical analyses were conducted at the biological replicate level to capture biological variability and support reproducible between-stage comparisons. The overall analytical and visualization workflow adopted in this study follows the same structured approach previously applied in related metabolomics investigations by Ogofure et al [1], allowing for methodological consistency and direct comparison across studies [9,19]. Initial overlap and exclusivity of metabolites between developmental stages were visualized using a scaled Venn diagram, which clearly distinguished GFC specific, RFC specific, and shared metabolites, with paired differences statistically evaluated using McNemar’s test. Overall differences in metabolite distribution were further quantified using a bar chart summarizing unique and shared metabolites, accompanied by a Chi-square test to assess the association between fruit coat stage and metabolite occurrence. To evaluate whether metabolite specialization was biased toward a particular stage, a binomial test was used to compare the number of RFC specific and GFC specific metabolites, with results displayed alongside a proportional pie chart. Compound class level patterns were examined through stacked bar plots of the top compound types, integrating Fisher’s exact test to identify compound classes significantly associated with either fruit coat stage. A complementary paired comparison of compound type abundance between GFC and RFC was performed using a Wilcoxon signed rank test and visualized using grouped horizontal bar charts. These visualizations and statistical analyses provide a robust, multi-level assessment of developmental shifts in metabolite composition, supporting stage-dependent metabolic differentiation between green and ripe fruit coats.

## Limitations

We acknowledge that the metabolite annotations are classified as Level 2 confidence (putative annotation) according to MSI guidelines, as analytical standards were not used for confirmation. The extraction solvent (methanol:chloroform 1:1) favours mid-polar and non-polar metabolites, potentially underrepresenting highly polar compounds. In particular, highly polar primary metabolites, such as mono- and disaccharides, were not fully captured under the present analytical conditions. Comprehensive profiling of these compounds would require dedicated polar metabolomics workflows (e.g., aqueous extraction coupled with Hydrophilic Interaction Liquid Chromatography-Mass Spectrometry (HILIC-LC-MS) or derivatization-based Gas Chromatography-Mass Spectrometry (GC–MS)). In addition, although samples were visually monitored during washing and no pigment leaching was observed, minor loss of highly polar or water-soluble metabolites during the washing and drying procedures cannot be completely excluded. Furthermore, the dataset represents a single geographic location and collection time point, as seasonal and environmental factors may influence metabolite profiles in the plant. The quantitative comparisons between ripe and green fruit coats are based on normalized peak areas rather than absolute quantification with internal standards. Lastly, while the MS/MS fragmentation patterns support proposed structures, definitive structural elucidation would require isolation and NMR spectroscopy. Therefore, these limitations can be addressed in future studies where we propose that the targeted metabolite quantification should be done using standards, and exploring alternative extraction solvents to capture a broader metabolite spectrum. Furthermore, multi-site and multi-season sampling should be conducted to assess geographic and temporal variation in plant metabolomics, and integration with transcriptomic data should be used to link metabolite profiles with gene expression patterns in the plant. Finally, the isolation and structural characterization of key bioactive metabolites using preparative chromatography and NMR spectroscopy is encouraged. The number of metabolites annotated at the MS2 confidence level (MSI Level 2) is relatively limited, which is attributable to several factors inherent to untargeted LC-QTOF-MS/MS analysis of specialized plant metabolites: (i) the incompleteness of publicly available MS2 spectral reference libraries for non-model plant secondary metabolites, particularly uncommon alkaloid and terpenoid subclasses; (ii) the low fragmentation efficiency of glycosylated steroidal alkaloids at the applied collision energy of 7.0 eV; (iii) data-dependent acquisition (DDA) coverage limitations, which may preclude consistent MS2 acquisition for low-abundance precursor ions across all biological replicates; and (iv) the stringent cosine-similarity threshold (≥0.7) applied during library matching. Future studies should consider employing variable collision energy ramps (e.g., 10–40 eV), data-independent acquisition (DIA/AIF) methods, and expanded spectral libraries (e.g., GNPS, LOTUS) to improve MS2 annotation rates. Additionally, fruit maturation stages were classified using qualitative visual and tactile criteria (colour and pericarp texture) rather than quantitative firmness measurements. While the criteria were standardized and rigorously applied, the absence of instrument-based firmness data (such as penetrometer measurements) represents a limitation, and future studies should incorporate quantitative texture analysis to provide objective stage-classification metrics. Furthermore, no exogenous internal standard was added to the sample extracts; therefore, quantitative comparisons between maturation stages are based on normalized peak areas and do not reflect absolute metabolite concentrations. Incorporation of a structurally relevant stable isotope-labelled internal standard added at the extraction step is recommended for future studies requiring absolute quantification.

## Ethics Statement

This research involved collection of plant material from the University of Johannesburg campus with appropriate institutional permissions. No endangered or protected plant species were involved. A voucher specimen (BTNPSP02) has been deposited in the publicly accessible herbarium of the University of Johannesburg for future reference.

## CRediT Author Statement

**Abraham Goodness Ogofure:** Methodology, Formal analysis, Resources, Data curation, Visualization, Writing - original draft, Writing - review & editing. **Ezekiel Green:** Supervision, Conceptualization, Investigation, Resources, Project administration, Funding acquisition, Writing - review & editing.

## Data Availability

FigshareDataset on the Differential Metabolijte Composition of Ripe and Green Fruit Coats of Solanum mauritianum (Original data)

## Appendix Supplementary materials

Image, application 1

## References

[1] Ogofure A.G., Sebola T., Green E. (2025). Antibacterial and anticancer properties of Solanum mauritianum fruit components analyzed using LC-QTOF-MS/MS. Sci. Rep..

[2] Jayakumar K., Murugan K. (2016). Evaluation of major phytochemicals in the leaves and fruits of Solanum Mauritianum scop.: a potential herbal drug. Int. J. Res. Ayurveda Pharm..

[3] Quinet M., Angosto T., Yuste-Lisbona F.J., Blanchard-Gros R., Bigot S., Martinez J.P., Lutts S. (2019). Tomato fruit development and metabolism. Front. Plant Sci..

[4] Rodriguez-Perez C., Gomez-Caravaca A.M., Guerra-Hernandez E., Cerretani L., Garcia-Villanova B., Verardo V. (2018). Comprehensive metabolite profiling of solanum tuberosum L. (potato) leaves by HPLC-ESI-QTOF-MS. Food Res. Int..

[5] Tohge T., Alseekh S., Fernie A.R. (2013). On the regulation and function of secondary metabolism during fruit development and ripening. J. Exp. Bot..

[6] Afroz M., Akter S., Ahmed A., Rouf R., Shilpi J.A., Tiralongo E., Sarker S.D., Goransson U., Uddin S.J. (2020). Ethnobotany and antimicrobial peptides from plants of the Solanaceae Family: an update and future prospects. Front. Pharmacol..

[7] Wawrosch C., Zotchev S.B. (2021). Production of bioactive plant secondary metabolites through in vitro technologies-status and outlook. Appl. Microbiol. Biotechnol..

[8] Nakitto A.M.S., Byaruhanga Y.B., Wagner A.E., Muyonga J.H. (2023). Influence of ripeness stage on the bioactive compounds' contents and antioxidant activities of Solanum anguivi Lam. Fruits accessions. Heliyon.

[9] Ogofure A.G., Green E. (2025). Bioactivity and metabolic profiling of crude extracts from endophytic bacteria linked to Solanum mauritianum scope: discovery of antibacterial and anticancer properties. Heliyon.

[10] Zhao D.K., Zhao Y., Chen S.Y., Kennelly E.J. (2021). Solanum steroidal glycoalkaloids: structural diversity, biological activities, and biosynthesis. Nat. Prod. Rep..

[11] Liu L.Y., Peng Q., Yang Y.K., Wu T., Chen L.X., Lin L. (2025). Seven previously undescribed steroidal alkaloids from the fruit of Solanum nigrum L. and their biological activities. Phytochemistry.

[12] Bouslamti M., Metouekel A., Chelouati T., El Moussaoui A., Barnossi A.E., Chebaibi M., Nafidi H.A., Salamatullah A.M., Alzahrani A., Aboul-Soud M.A.M., Bourhia M., Lyoussi B., Benjelloun A.S. (2022). Solanum elaeagnifolium Var. Obtusifolium (Dunal) Dunal: antioxidant, antibacterial, and antifungal activities of polyphenol-rich extracts chemically characterized by use of In vitro and In silico approaches. Molecules.

[13] Al Sinani S.S.S., Eltayeb E.A. (2017). The steroidal glycoalkaloids solamargine and solasonine in Solanum plants. S. Afr. J. Bot..

[14] Carrari F., Baxter C., Usadel B.r., Urbanczyk-Wochniak E., Zanor M.-I., Nunes-Nesi A., Nikiforova V., Centero D., Ratzka A., Pauly M., Sweetlove L.J., Fernie A.R. (2006). Integrated analysis of metabolite and transcript levels reveals the metabolic shifts that underlie tomato fruit development and highlight regulatory aspects of metabolic network behavior. Plant Physiol..

[15] Hu C., Gao X., Dou K., Zhu C., Zhou Y., Hu Z. (2023). Physiological and metabolic changes in Tamarillo (Solanum betaceum) during fruit ripening. Molecules.

[16] Moco S., Capanoglu E., Tikunov Y., Bino R.J., Boyacioglu D., Hall R.D., Vervoort J., De Vos R.C. (2007). Tissue specialization at the metabolite level is perceived during the development of tomato fruit. J. Exp. Bot..

[17] Hasan K., Sabiha S., Islam N., Pinto J.F., Silva O. (2024). Ethnomedicinal usage, phytochemistry and pharmacological potential of Solanum surattense Burm. F. Pharm..

[18] Sumner L.W., Amberg A., Barrett D., Beale M.H., Beger R., Daykin C.A., Fan T.W., Fiehn O., Goodacre R., Griffin J.L., Hankemeier T., Hardy N., Harnly J., Higashi R., Kopka J., Lane A.N., Lindon J.C., Marriott P., Nicholls A.W., Reily M.D., Thaden J.J., Viant M.R. (2007). Proposed minimum reporting standards for chemical analysis chemical analysis working group (CAWG) metabolomics standards initiative (MSI). Metabolomics.

[19] Ogofure A.G., Sebola T., Green E. (2025). Metabolomic profile and bioactivity of fungal endophytes isolated from Crinum macowanii. BMC Complem. Med. Ther..

