# Supplementary material for: Dataset on the differential metabolite composition of ripe and green fruit coats of Solanum mauritianum
Source: Data Brief. 2026 May 7;66:112829. doi: 10.1016/j.dib.2026.112829 (PMC13195779; doi:10.1016/j.dib.2026.112829)
Supplement: Supplementary file 1 [file mmc1.docx]

Supplementary Figure 1: Extracted ion chromatogram (EIC) of secondary metabolites from *Solanum mauritianum* ripe fruits coat – S1 RFC (left) and their MS^2^ fragments (right).

| **EIC: y axis = Intensity; x axis = Time (min)** | **MS^2^: y axis = Intensity; x axis = *m/z* (Mass to charge ratio)** |
| --- | --- |
| Base Peak Chromatogram  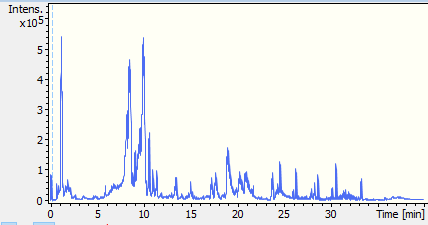 | 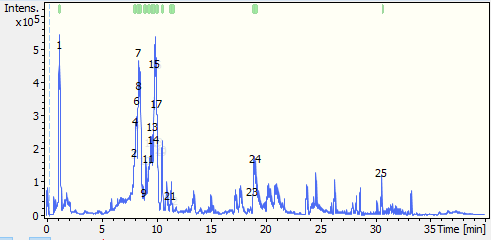 |
| Cardiospermin  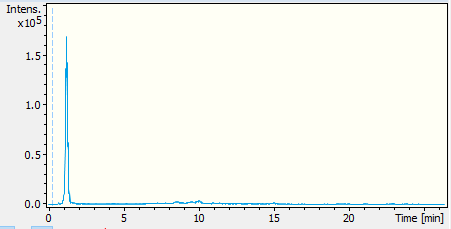  **EIC (*m/z* 276.0970; +MS)** | 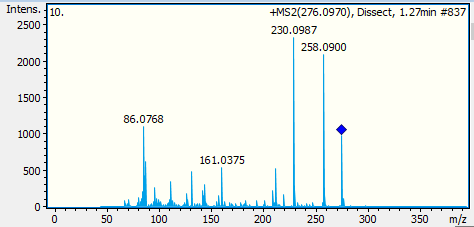  **Fragments *m/z* 276.0970; +MS/MS** |
| Furofoline I  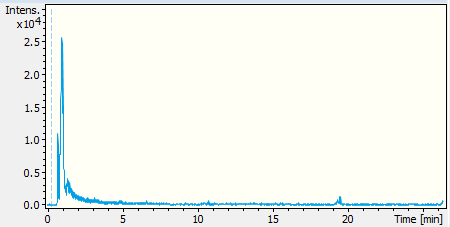  **EIC (*m/z* 266.0796; +MS)** | 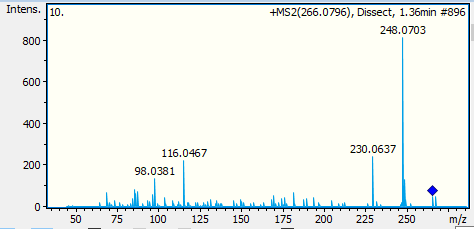  **Fragments *m/z* 266.0796; +MS/MS** |
| Riccionidin A  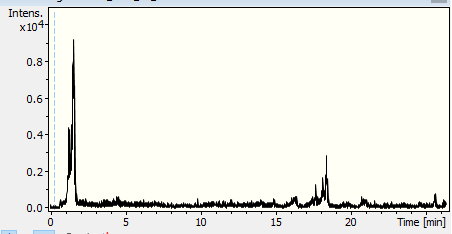  **EIC (*m/z* 286.0453; +MS)** | 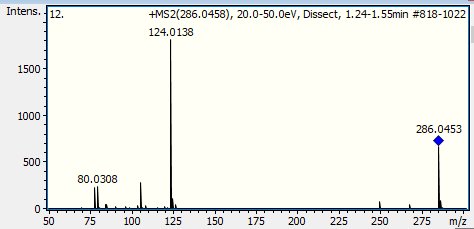  **Fragments *m/z* 286.0453; +MS/MS** |
| Bergenin  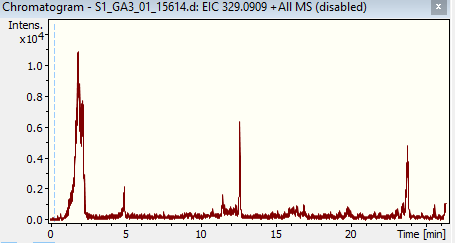  **EIC (*m/z* 329.0909; +MS)** | 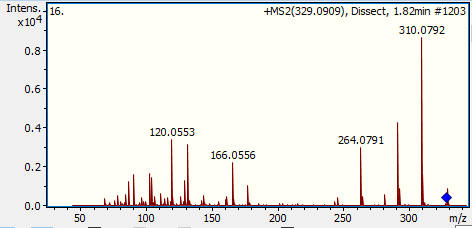  **Fragments *m/z* 329.0909; +MS/MS** |
| Cusparine  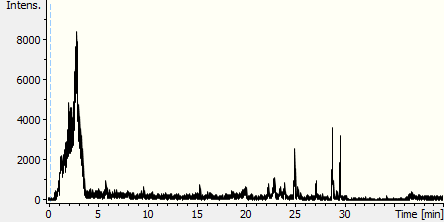  **EIC (*m/z* 308.1209; +MS)** | 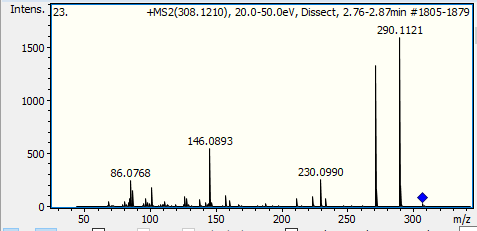  **Fragments *m/z* 308.1209; +MS/MS** |
| Theogallin  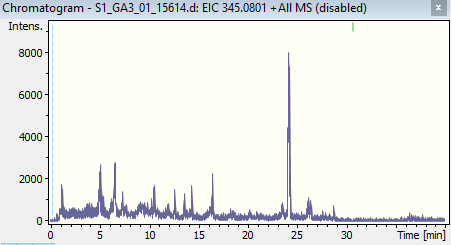  **EIC (*m/z* 345.0801; +MS)** | 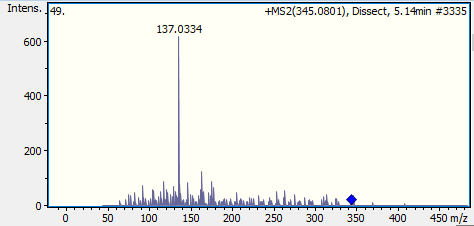  **Fragments *m/z* 345.0801; +MS/MS** |
| Homoeriodictyol chalcone  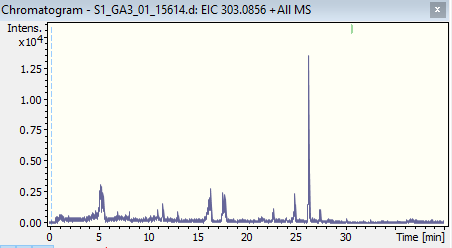  **EIC (*m/z* 303.0856; +MS)** | 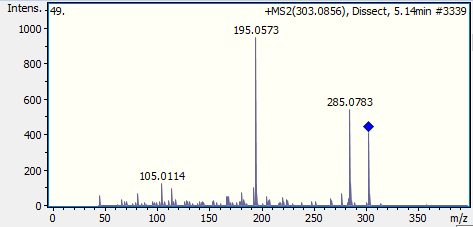  **Fragments *m/z* 303.0856; +MS/MS** |
| Berberastine  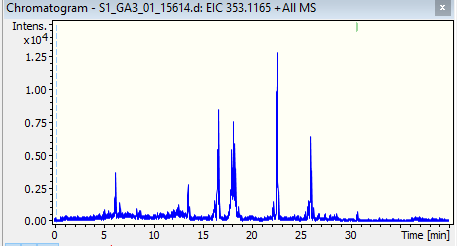  **EIC (*m/z* 353.1165; +MS)** | 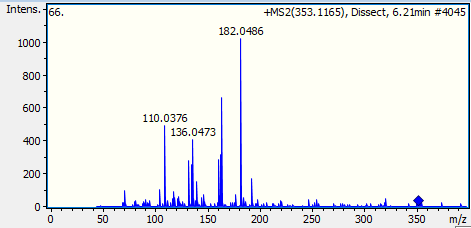  **Fragments *m/z* 353.1165; +MS/MS** |
| Scopoletin  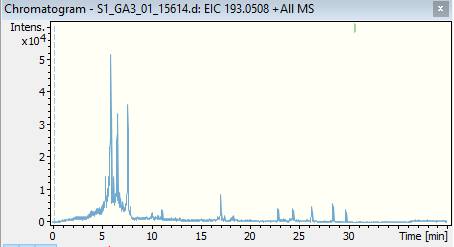  **EIC (*m/z* 193.0508; +MS)** | 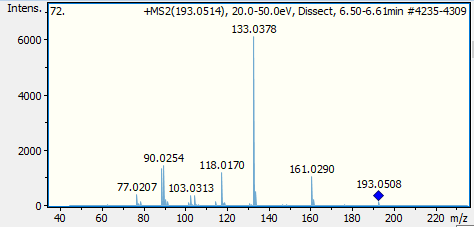  **Fragments *m/z* 193.0508; +MS/MS** |
| Lysergic acid  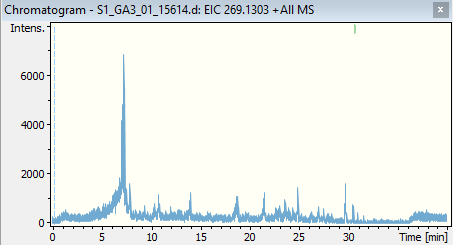  **EIC (*m/z* 269.1303; +MS)** | 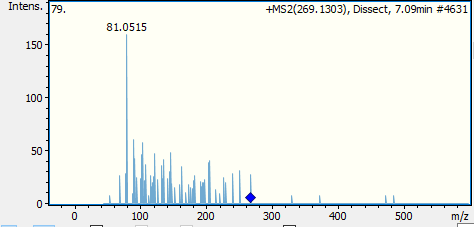  **Fragments *m/z* 269.1303; +MS/MS** |
| Glycophymoline  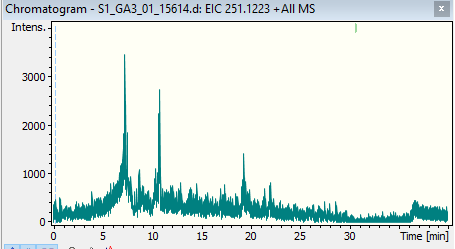  **EIC (*m/z* 251.1223; +MS)** | 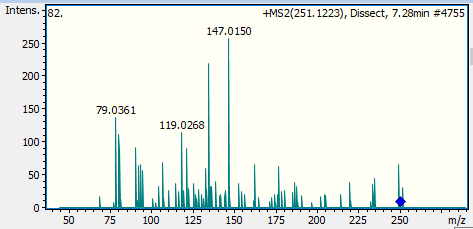  **Fragments *m/z* 251.1223; +MS/MS** |
| Solasonine  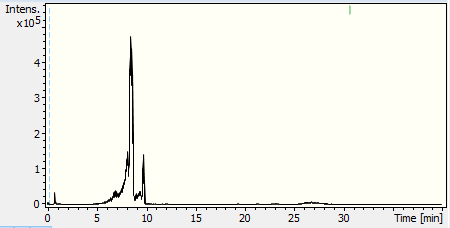  **EIC (*m/z* 884.3903; +MS)** | 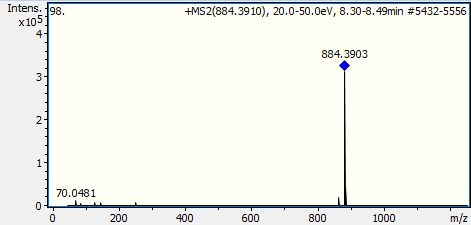  **Fragments *m/z* 884.3903; +MS/MS** |
| α-Solanine  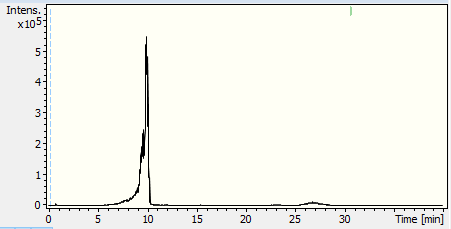  **EIC (*m/z* 868.3953; +MS)** | 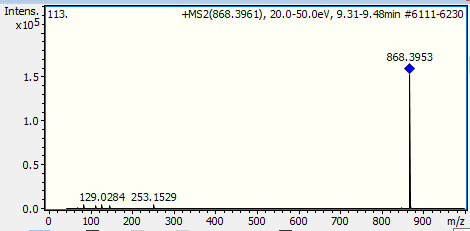  **Fragments *m/z* 868.3953; +MS/MS** |
| Imperialine  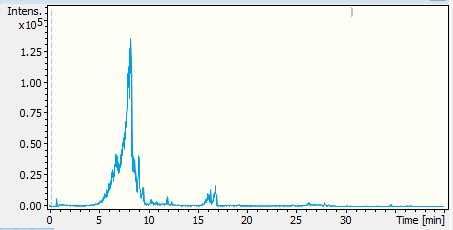  **EIC (*m/z* 430.2686; +MS)** | 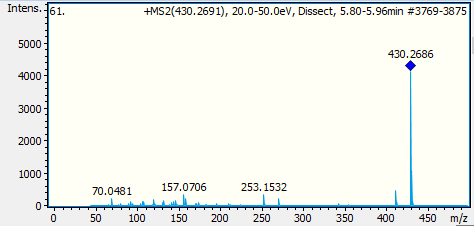  **Fragments *m/z* 430.2686; +MS/MS** |
| Genipin  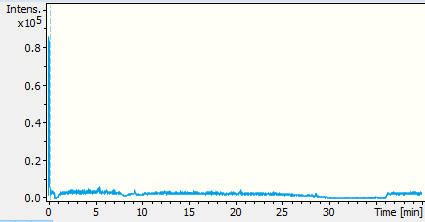  **EIC (*m/z* 227.1021; +MS)** | 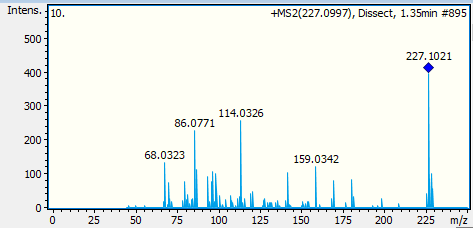  **Fragments *m/z* 227.1021; +MS/MS** |
| Solasodine  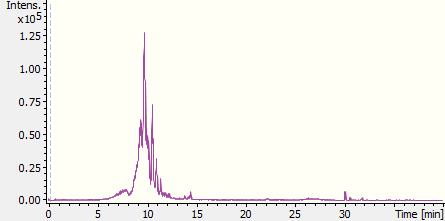  **EIC (*m/z* 414.2751; +MS)** | 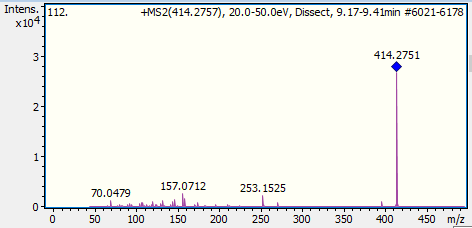  **Fragments *m/z* 414.2751; +MS/MS** |
| α-Ergocryptine  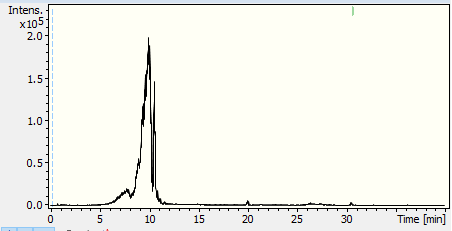  **EIC (*m/z* 576.3100; +MS)** | 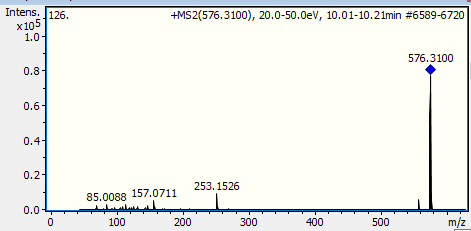  **Fragments *m/z* 576.3100; +MS/MS** |
| Callicarpone  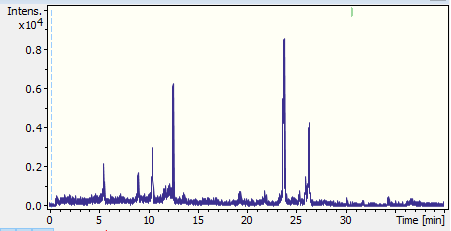  **EIC (*m/z* 333.2115; +MS)** | 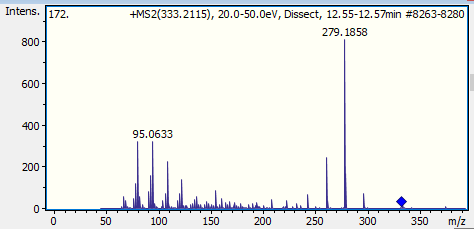  **Fragments *m/z* 333.2115; +MS/MS** |
| 10-Deoxysarpagine  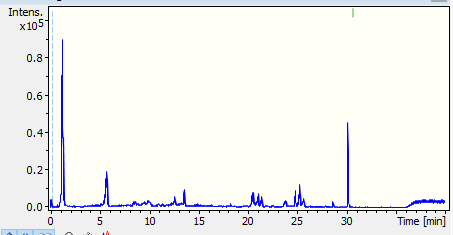  **EIC (*m/z* 295.1791; +MS)** | 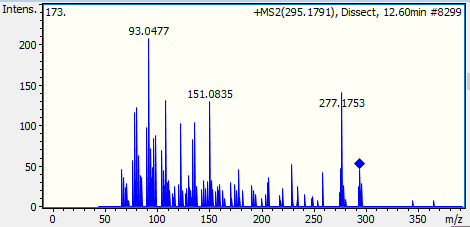  **Fragments *m/z* 295.1791; +MS/MS** |
| Montanol  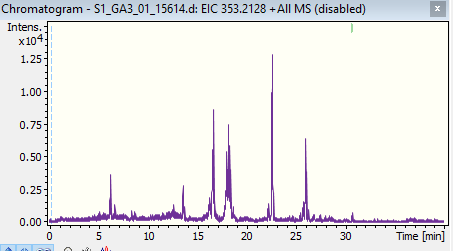  **EIC (*m/z* 353.2128; +MS)** | 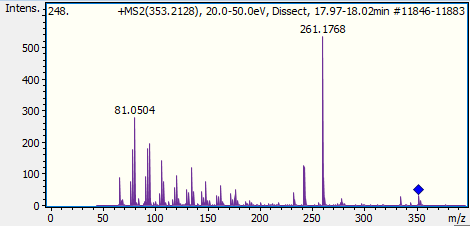  **Fragments *m/z* 353.2128; +MS/MS** |
| Hypercalin B  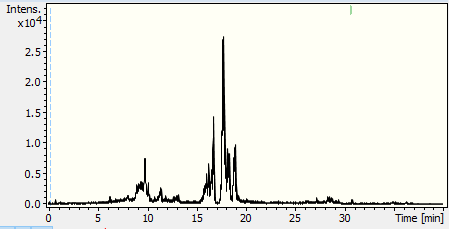  **EIC (*m/z* 519.3100; +MS)** | 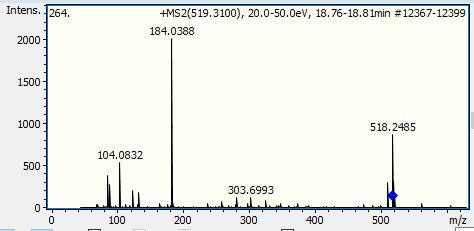  **Fragments *m/z* 519.3100; +MS/MS** |
| Eugenin  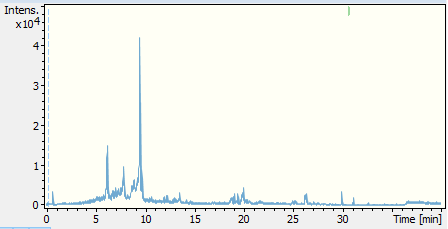  **EIC (*m/z* 207.0642; +MS)** | 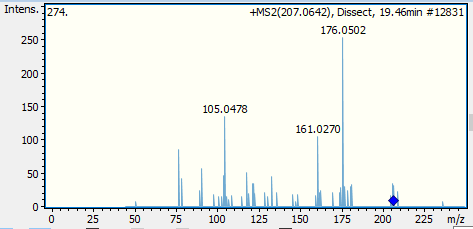  **Fragments *m/z* 207.0642; +MS/MS** |
| Thalicarpine  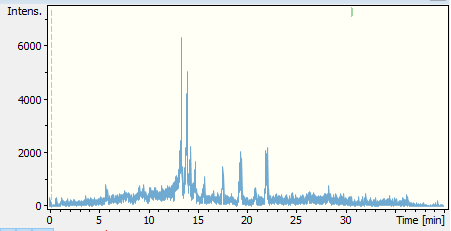  **EIC (*m/z* 697.3305; +MS)** | 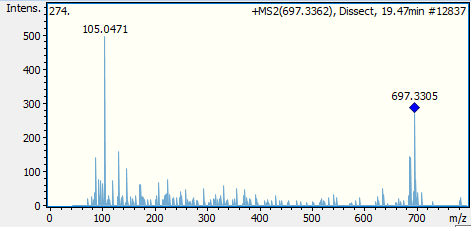  **Fragments *m/z* 697.3305; +MS/MS** |
| Anatabine  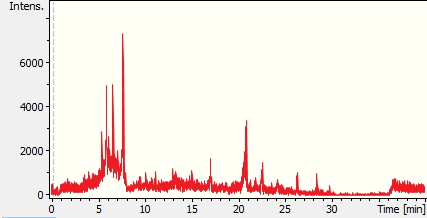  **EIC (*m/z* 161.0641; +MS)** | 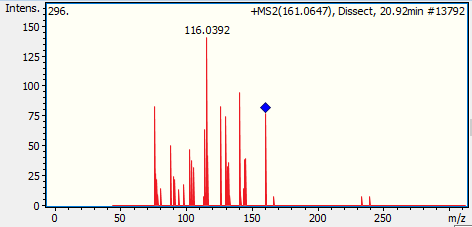  **Fragments *m/z* 161.0641; +MS/MS** |
| Ibogamine  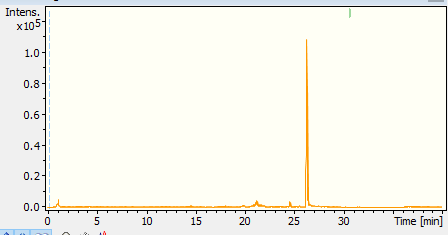  **EIC (*m/z* 281.1998; +MS)** | 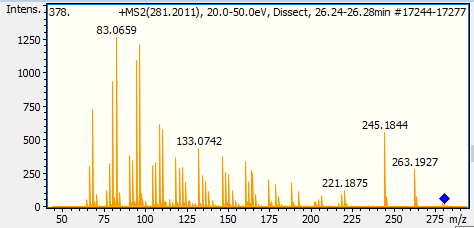  **Fragments *m/z* 281.1998; +MS/MS** |
| Ibogaine  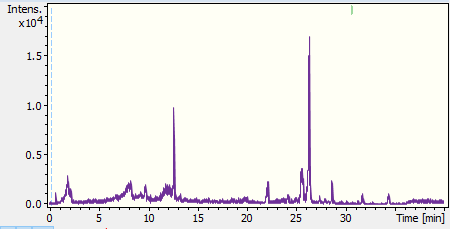  **EIC (*m/z* 311.2077; +MS)** | 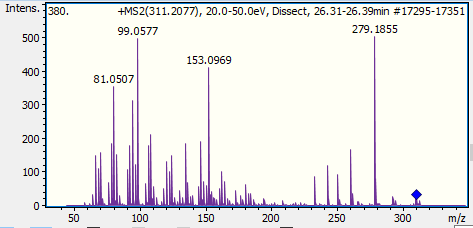  **Fragments *m/z* 311.2077; +MS/MS** |
| Loganin  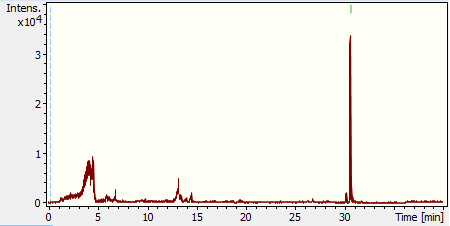  **EIC (*m/z* 391.1497; +MS)** | 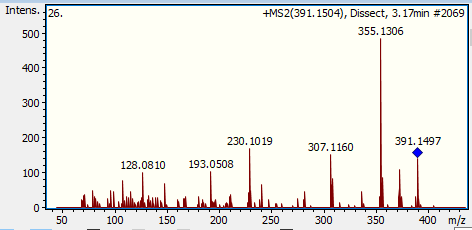  **Fragments *m/z* 391.1497; +MS/MS** |
| Tingenone  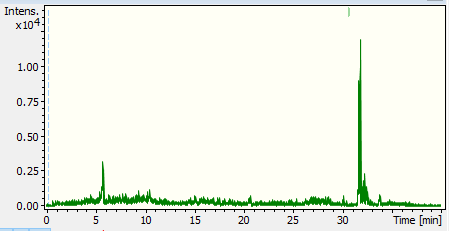  **EIC (*m/z* 421.2636; +MS)** | 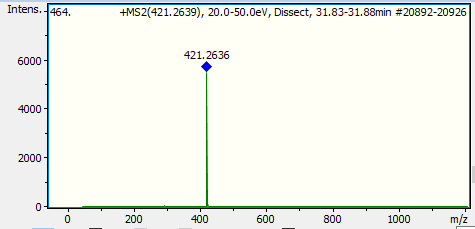  **Fragments *m/z* 421.2636; +MS/MS** |

Supplementary Figure 2: Extracted ion chromatogram (EIC) of secondary metabolites from *Solanum mauritianum* unripe (green) fruits coat – S4 GFC (left) and their MS^2^ fragments (right).

| **EIC: y axis = Intensity; x axis = Time (min)** | **MS^2^: y axis = Intensity; x axis = *m/z* (Mass to charge ratio)** |
| --- | --- |
| Base Peak Chromatogram (BPC)  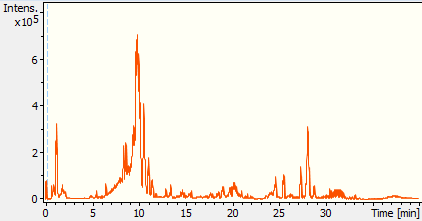 | 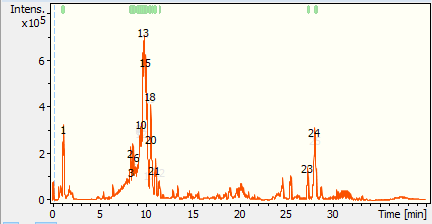 |
| Cardiospermin  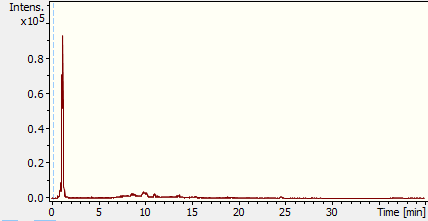  **EIC (*m/z* 276.0961; +MS)** | 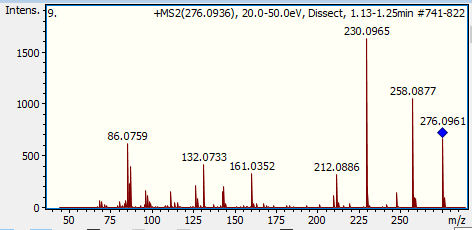  **Fragments *m/z* 276.0961; +MS/MS** |
| Vicine  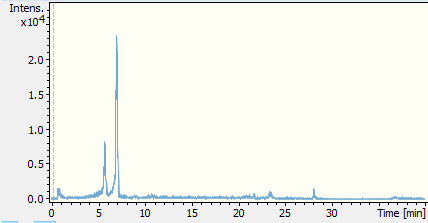  **EIC (*m/z* 305.1069; +MS)** | 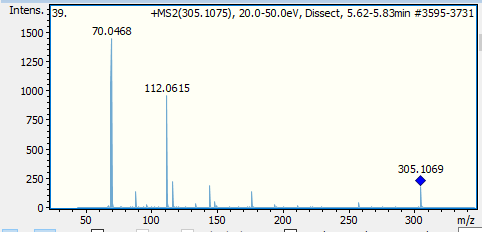  **Fragments *m/z* 305.1069; +MS/MS** |
| Scopoletin  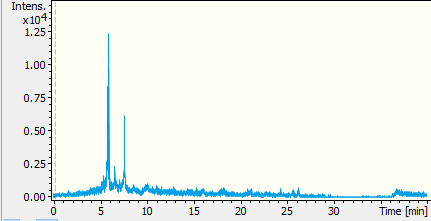  **EIC (*m/z* 193.0514; +MS)** | 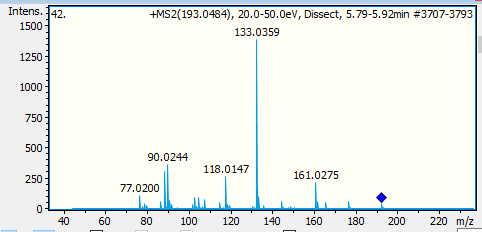  **Fragments *m/z* 193.0514; +MS/MS** |
| Solasonine  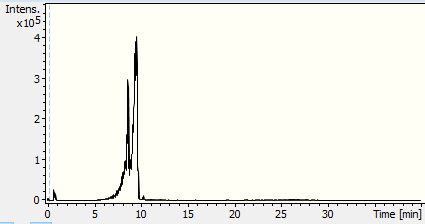  **EIC (*m/z* 884.3910; +MS)** | 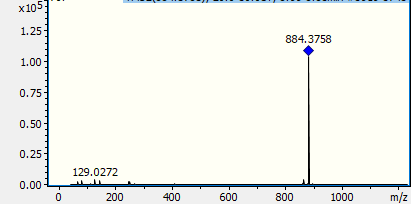  **Fragments *m/z* 884.3910; +MS/MS** |
| α-Solanine  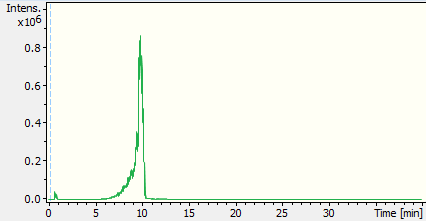  **EIC (*m/z* 868.3911; +MS)** | 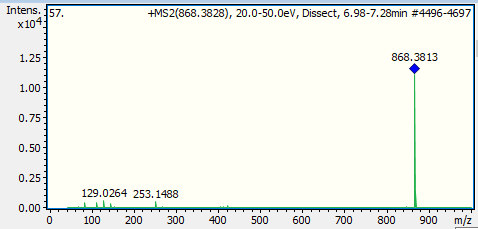  **Fragments *m/z* 868.3911; +MS/MS** |
| Imperialine  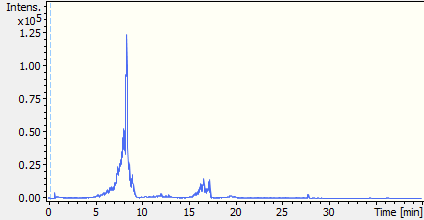  **EIC (*m/z* 430.2621; +MS)** | 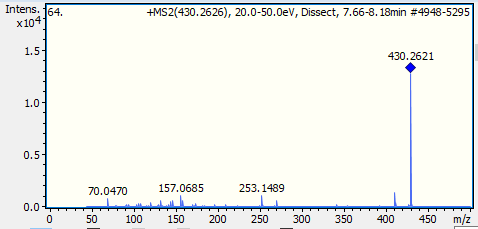  **Fragments *m/z* 430.2621; +MS/MS** |
| α-Ergocryptine  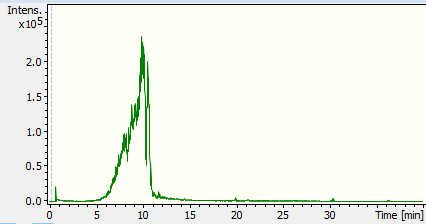  **EIC (*m/z* 576.3015; +MS)** | 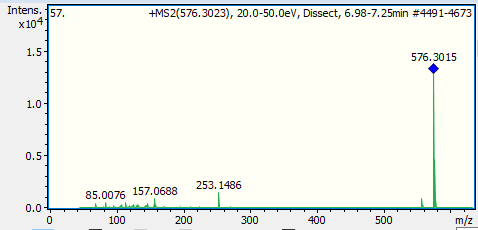  **Fragments *m/z* 576.3015; +MS/MS** |
| Solasodine  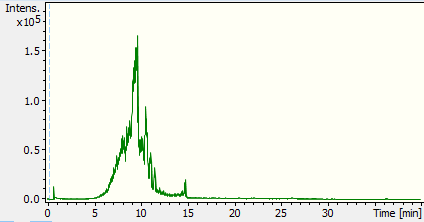  **EIC (*m/z* 414.2679; +MS)** | 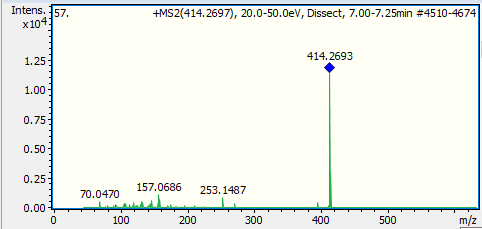  **Fragments *m/z* 414.2679; +MS/MS** |
| Callicarpone  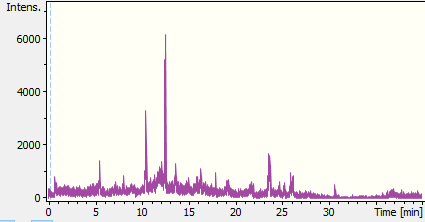  **EIC (*m/z* 333.2115; +MS)** | 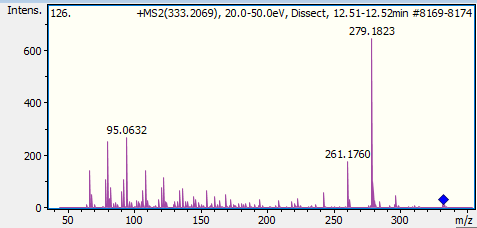  **Fragments *m/z* 333.2115; +MS/MS** |
| Montanol  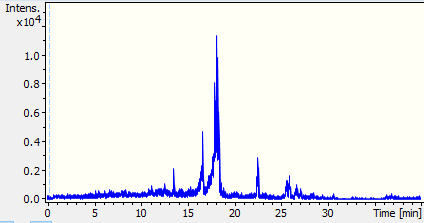  **EIC (*m/z* 353.2128; +MS)** | 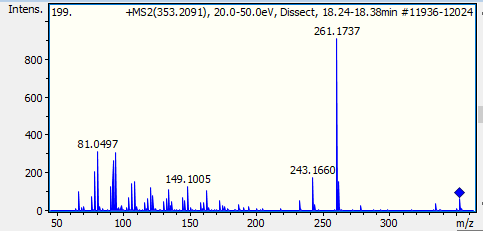  **Fragments *m/z* 353.2128; +MS/MS** |
| Coniferyl alcohol  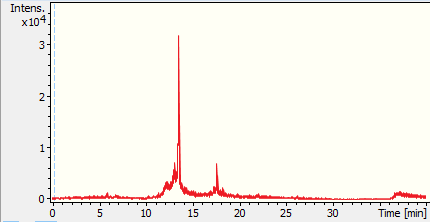  **EIC (*m/z* 181.0869; +MS)** | 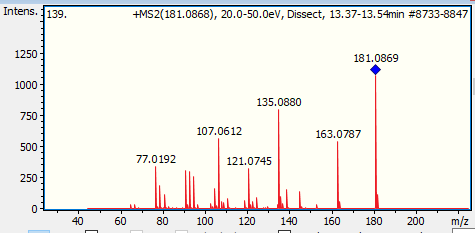  **Fragments *m/z* 181.0869; +MS/MS** |
| Anatabine  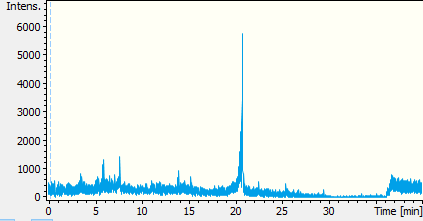  **EIC (*m/z* 161.0641; +MS)** | 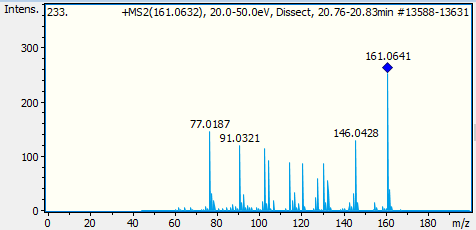  **Fragments *m/z* 161.0641; +MS/MS** |
| Lycocernuine  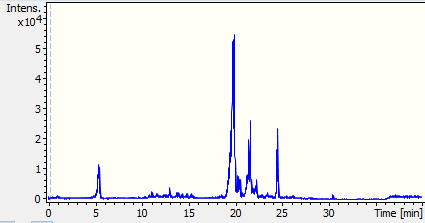  **EIC (*m/z* 279.1819; +MS)** | 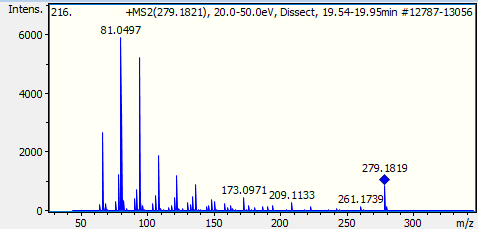  **Fragments *m/z* 279.1819; +MS/MS** |
| Diosmin  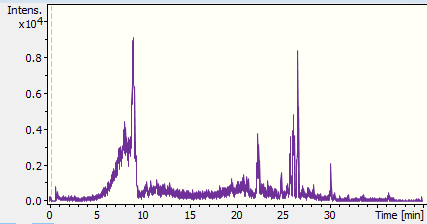  **EIC (*m/z* 609.1812; +MS)** | 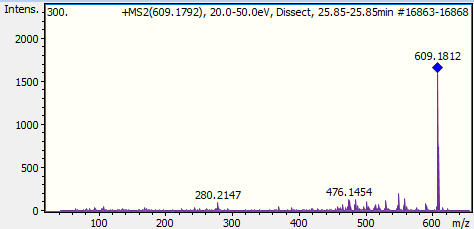  **Fragments *m/z* 609.1812; +MS/MS** |
| Cannabielsoin  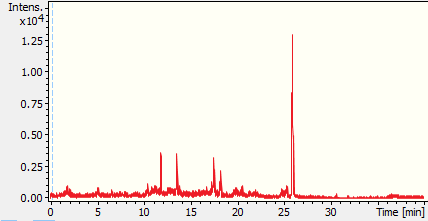  **EIC (*m/z* 331.2300; +MS)** | 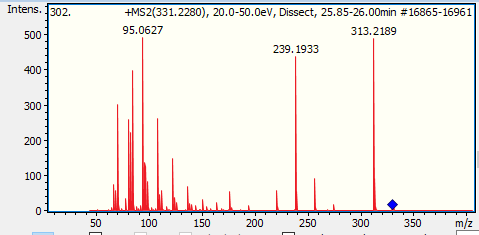  **Fragments *m/z* 331.2300; +MS/MS** |
| Ibogamine  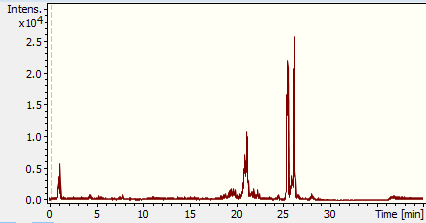  **EIC (*m/z* 281.1998; +MS)** | 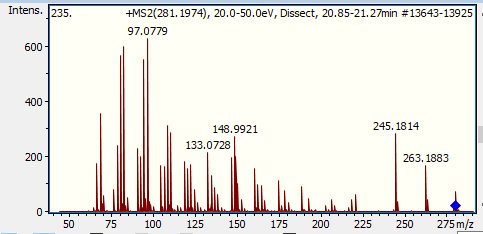  **Fragments *m/z* 281.1998; +MS/MS** |
| Isoswertisin 2''-rhamnoside  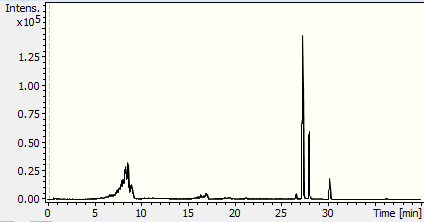  **EIC (*m/z* 593.1855; +MS)** | 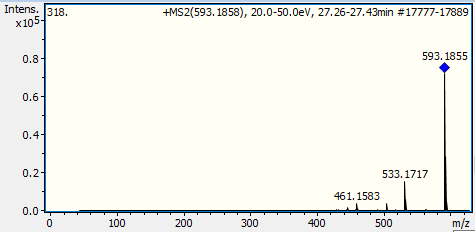  **Fragments *m/z* 593.1855; +MS/MS** |
| Absinthin  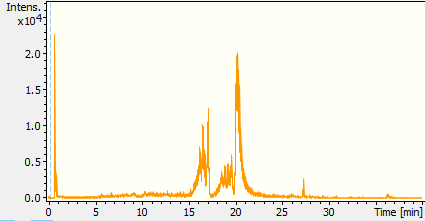  **EIC (*m/z* 497.2867; +MS)** | 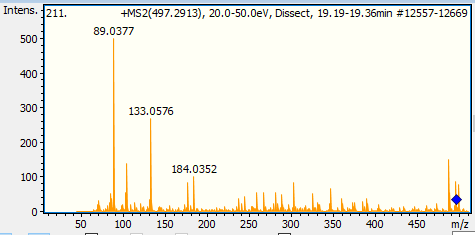  **Fragments *m/z* 497.2867; +MS/MS** |
